# Unexpected Reduced Biventricular Ejection Fraction in a Healthy Young Male

**DOI:** 10.7759/cureus.7292

**Published:** 2020-03-16

**Authors:** Alejandro Sanchez-Nadales, Valentina Celis, Miguel Treminio Quezada, Jessica Navarro, Elena Caldeira

**Affiliations:** 1 Internal Medicine, Advocate Illinois Masonic Medical Center, Chicago, USA; 2 Internal Medicine, Mount Sinai Medical Center, Miami, USA

**Keywords:** heart failure with reduced ejection fraction, viral myocarditis, endomyocardial biopsy, cardiac magnetic resonance, transthoracic echocardiography

## Abstract

We describe the case of a 31-year-old male who came to the emergency department complaining of marked bilateral lower extremities edema, dyspnea, fatigue, and exertion intolerance. Strategies for the management of viral myocarditis with acute heart failure include pharmacological therapies and mechanical circulatory assist devices if required. Despite multiple available diagnostic methods and treatments, viral myocarditis remains as an etiology of challenging diagnosis, and poor prognosis with a high mortality rate.

## Introduction

Heart failure with reduced ejection fraction (HFrEF) results in a decreased ability of the heart to pump blood to the rest of the body, resulting in an insufficient amount of oxygenated blood reaches the organs to fulfill their metabolic demands. The most common causes of HFrEF include coronary artery disease, valvular heart disease, and hypertension [1]. However, other etiologies for HFrEF needs to be considered when the cause is not clear. Myocarditis (an inflammatory condition of heart muscle) is a relevant cause of dilated cardiomyopathy worldwide, with viral infections been accountable for the vast majority of cases [2]. Heart failure (HF) usually presents with dyspnea at exertion or rest, fatigue, lower extremity edema, orthopnea, and paroxysmal nocturnal dyspnea. Signs include evidence of volume overload (elevated jugular venous pressure, peripheral edema, and pulmonary rales) or diminished perfusion (cold extremities in severe cases) [3]. The prevalence of HFrEF increases with age [4]. Initial workup should include a 12-lead electrocardiogram (ECG), a chest x-ray, complete blood count, serum chemistries, fasting lipid profile, liver function tests, thyroid-stimulating hormone, and B-type natriuretic peptide (BNP). Transthoracic echocardiography (TTE) is used to assess left ventricular ejection fraction (LVEF) and confirm the diagnosis of HFrEF. It is crucial to consider testing patients for specific conditions in whom the underlying cause of heart failure has not been identified. In patients that experienced typical flu-like symptoms and heart failure symptoms the diagnosis of viral myocarditis should be included highly in the list of differential diagnosis. In that scenario, cardiac magnetic resonance (CMR) and endomyocardial biopsy (EMB) should be included as part of the workup. Finally, the use of a wireless implantable hemodynamic monitoring device in conjunction with a heart failure specialist should be considered in selected patients with HFrEF who remain symptomatic despite standard medical therapy [5, 6]. 

## Case presentation

**Figure 1:**
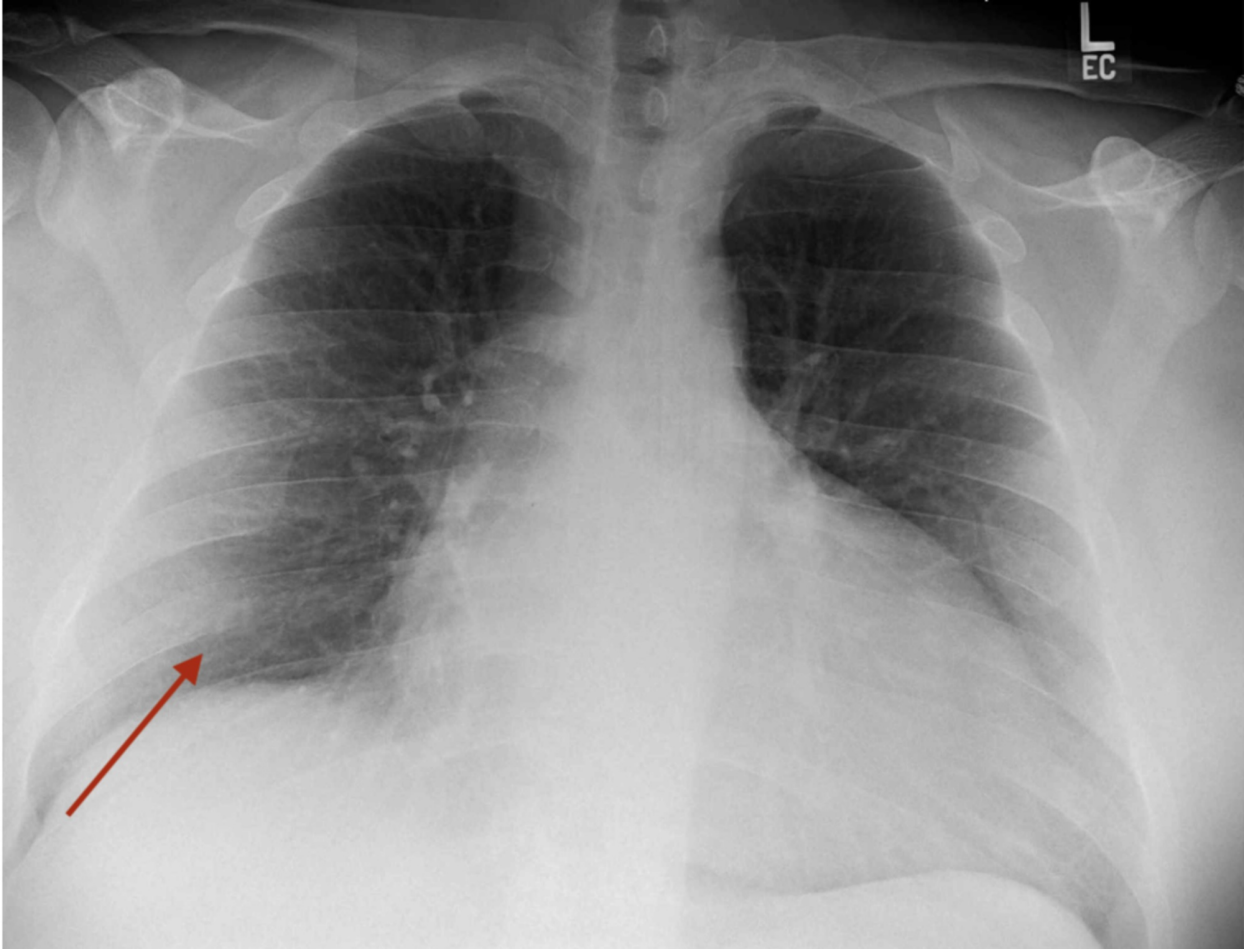
Chest X-ray Enlarge cardiac silhouette and mild cephalization of the pulmonary vasculature.

**Figure 2:**
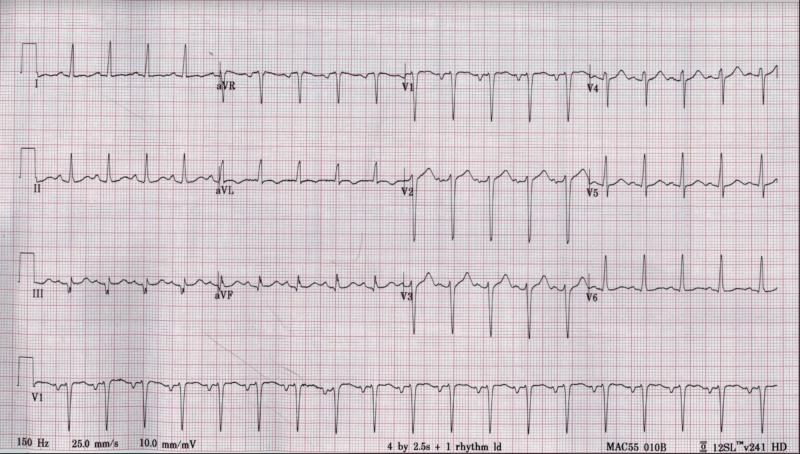
12-lead electrocardiogram

A 31-year-old male, without any significant past medical history, who came to the emergency department with sudden onset paroxysmal nocturnal dyspnea, complete intolerance to exertion along with marked bilateral lower extremities edema (pitting). He reported having flu-like symptoms two weeks before presentation, symptoms included generalized weakness, non-productive cough, subjective fevers, chills, and sore throat, which self-resolved after five days. He stated that he had been in contact with multiple sick individuals with similar symptoms at work. He endorsed this is the first time with these complaints and tried over-the-counter medications for the common cold (including acetaminophen and anti-histaminic). Family history is only significant for essential hypertension. He is an active smoker (10 cigarettes daily for the past five years) and reported occasional marijuana consumption. Physical examination is remarkable for decreased peripheral pulses, bibasilar lung crackles, jugular venous distention, and cold extremities, and elevated blood pressure (210/140). Laboratory exams pertinent for hypokalemia at 3.1 mmol/L, mildly elevated creatinine at 1.31 mg/dL, elevated troponin I at 1.05 ng/mL, and marked raised N-terminal pro b-type natriuretic peptide (NT-pro-BNP) at 3097 pg/mL. The chest X-ray showed mild vascular congestion (Figure 1), and the electrocardiogram (EKG) proved sinus tachycardia without ischemic changes (Figure 2). Surprisingly, 2D transthoracic echocardiogram (TTE) revealed dilated chambers, severely depressed ejection fraction (estimated at 10-15%) with global hypokinesia, grade 2 diastolic dysfunction, high pulmonary pressure (55 mmHg), and moderate mitral valve regurgitation (Videos 1, 2). In the beginning, the differential diagnosis included coronary artery disease, viral myocarditis, and secondary causes of hypertension with hypokalemia like hyperaldosteronism with/without hyperreninemia pathologies (like adrenal hyperplasia, adrenal adenomas, proximal renal tubular acidosis, and renovascular diseases). The patient's elevated blood pressure was managed with labetalol, and he was transferred to the telemetry unit on continuous furosemide infusion. Throughout the next 24 hours, the patient remained tachycardic and severely hypertensive, despites several oral and intra-venous anti-hypertensive medications, the reason why nitroglycerin infusion was initiated, and he moved to the medical intensive care unit (MICU). 

**Video 1:** 2D transthoracic echocardiogram (parasternal long-axis view) The left ventricle is dilated with concentric hypertrophy and estimated left ventricular ejection fraction (LVEF) at 10-15%. Severe global hypokinesia and moderate mitral regurgitation.

**Video 2:** 2D transthoracic echocardiogram (apical four chambers view) Severely dilated right and left cavities. Global hypokinesia and left ventricular ejection fraction (LVEF) estimated at 10-15%.

**Figure 3:**
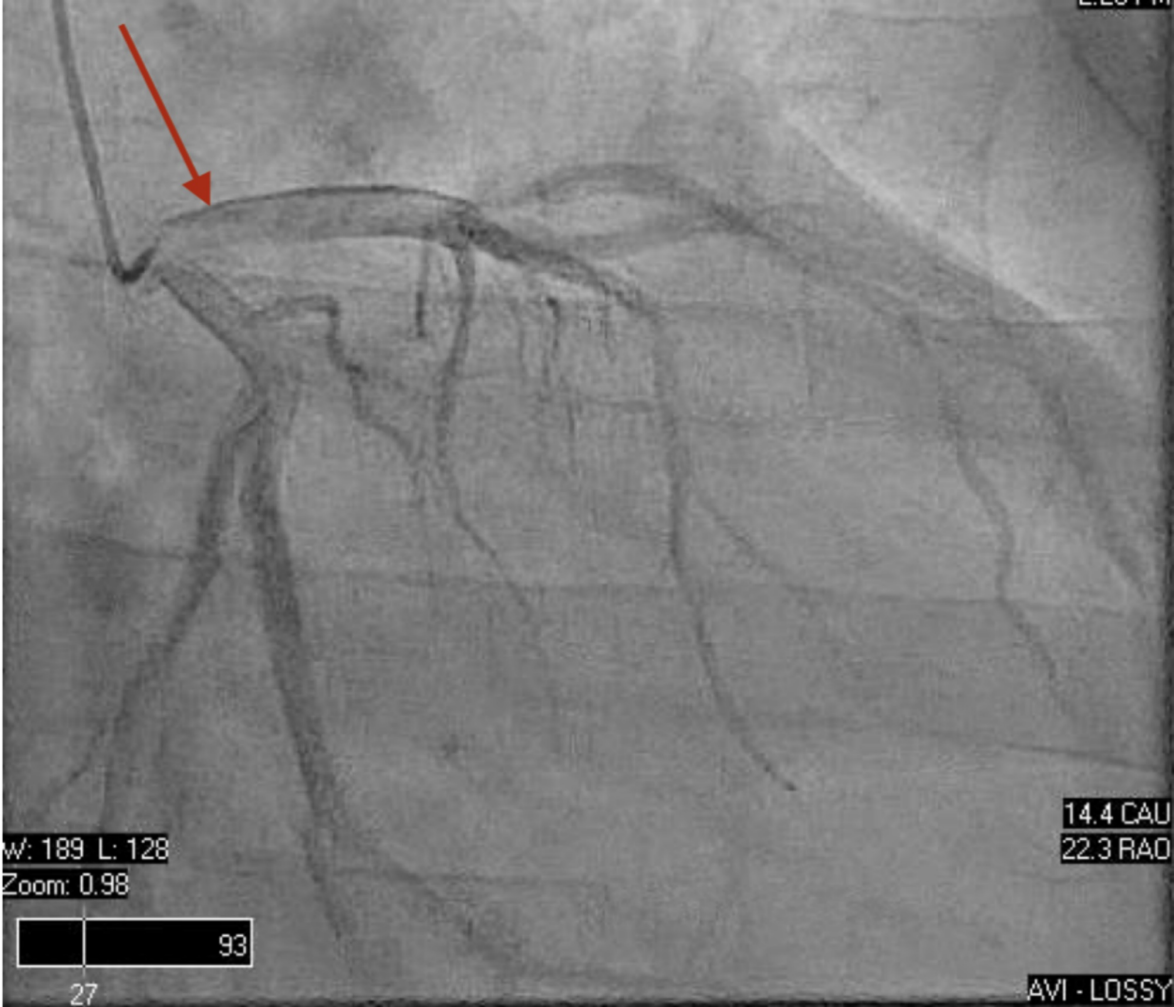
Left heart catheterization (left coronary angiography) Angiographically normal left main coronary artery, left anterior descending artery and left circumflex artery.

**Figure 4:**
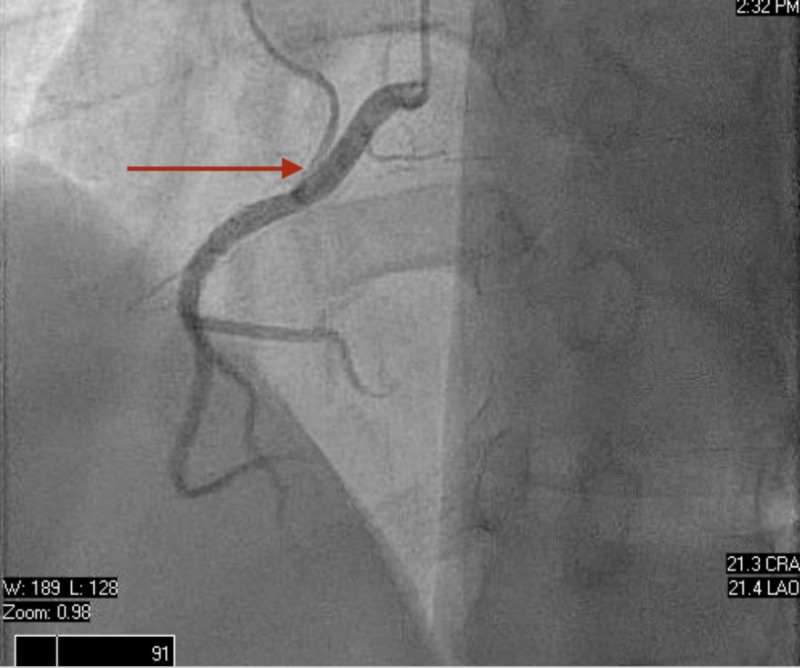
Left heart catheterization (right coronary angiography) Angiographically normal right coronary artery.

Secondary causes of hypertension were ruled out with typical values of aldosterone/renin activity ratio, cortisol level, and unremarkable bilateral renal arterial doppler. Right heart catheterization was performed, reporting hemodynamic values as follows: mean pulmonary capillary wedge pressure (PCW) at 24 mmHg, cardiac output of 3 L/min, and cardiac index of 2 L/min/m2. The left heart catheterization revealed angiographically normal coronary arteries (Figures 3 and 4), ruling out a cause of ischemic cardiomyopathy. An endomyocardial biopsy (EMB) collected during the heart catheterization revealed active lymphocytic myocarditis. During the next days at MICU, he developed lactic acidosis and respiratory distress requiring mechanical ventilation and an intra-aortic balloon pump for the management of suspected cardiogenic shock. The patient started to improve after two days in the MICU with constant monitoring and medical management, including carvedilol 12.5 mg twice daily and enalapril 10 mg twice daily, along with circulatory and ventilatory support, demonstrated by decreased levels of venous lactic acid and improved LVEF at bedside 2D echocardiogram. Intra-aortic balloon pump and endotracheal tube were removed successfully, and a repeated TTE showed raised LVEF estimated in 30-35%, normal cavities sizes, and mild mitral regurgitation (Videos 3, 4). He was discharged on low dose beta-blockers and angiotensin-converting enzyme inhibitors (ACEi). The patient maintained close follow up at the heart failure clinic with a satisfactory improvement of symptoms over the next two weeks. Additionally, an outpatient cardiac magnetic resonance (CMR) with and without contrast reported severe left ventricular hypertrophy (LVH), no visible scars, mild global hypokinesia without regional wall motion abnormalities, increasing LVEF up to 45% and RVEF at 35% (Video 5). The polymerase chain reaction technique demonstrated elevated titles of adenovirus, which is the suspected causal agent to the date. Finally, an outpatient TTE performed four months after discharge revealed normal systolic function with estimated LVEF on 50-55% (Video 6).

**Video 3:** Follow-up 2D transthoracic echocardiogram (parasternal long-axis view) Left ventricle with concentric hypertrophy and improved left ventricular ejection fraction (LVEF) estimated at 30-35%.

**Video 4:** Follow-up 2D transthoracic echocardiogram (apical four chambers view) The right and left cavities are now mildly dilated. Raised left ventricular ejection fraction (30-35%) and improved mitral regurgitation (mild from moderate).

**Video 5:** Cardiac magnetic resonance (CMR) with contrast Severe left ventricular hypertrophy (LVH), no visible scars. Mild global hypokinesia without regional wall motion abnormalities. Increasing left ventricular ejection fraction (LVEF) up to 45% and right ventricular ejection fraction (RVEF) at 35%.

**Video 6:** Follow-up 2D transthoracic echocardiogram (apical view with contrast) Marked improvement with normal left ventricular ejection fraction (LVEF) now estimated at 50-55%. Made as an outpatient four months after discharge.

## Discussion

Viral myocarditis has been recognized as a cause of congestive heart failure for more than 50 years, but it is still a challenging disease to diagnose and treat [7, 8]. In our case, the clinical presentation on admission showed a constellation of signs and symptoms that might not be specific to viral myocarditis. Echocardiography is an essential tool in the diagnosis of myocardial dysfunction, able to exclude other causes of heart failure such as congenital heart diseases, valvulopathies, or pericardial pathologies. Classical findings include global hypokinesia, with or without pericardial effusion, a variable degree of myocardial dilation, and atrioventricular regurgitation [9]. 

In the last few years, advances in noninvasive techniques such as CMR have been very useful in supporting the diagnosis of myocarditis. Toxic, infectious-inflammatory, infiltrative, or autoimmune processes occur at a cellular level, and only endomyocardial biopsy can establish the nature of the etiological agent [10-12]. In the study done by Philipp Lurz et al., only 50% of the patients with EMB-proven myocarditis showed pericardial effusion on cardiac magnetic resonance (CMR) at the time of hospital admission, which is consistent with this case report [13]. Endomyocardial biopsy is usually reserved for patients with acute dilated cardiomyopathy associated with hemodynamic compromise, life-threatening arrhythmia, and in those whose condition does not respond to conventional supportive therapy [2]. 

The long term prognosis in patients with acute heart failure secondary to acute myocarditis is debatable [14-15]. McCarthy et al. observed that patients with fulminant myocarditis who survive from the acute phase with aggressive hemodynamic support, are most likely to have a complete recovery of left ventricular function, with an excellent long term prognosis, compared with patients with acute non-fulminant myocarditis [16]. However, other authors also have described that a reduced left ventricular ejection fraction at onset is the main predictor for survival. Despite having an abrupt decline in cardiac function, our patent had an excellent recovery. The patient's age, supplementing with the non-presence of comorbidities, and the proper interventions might be contributors to his long-term recovery. 

## Conclusions

Viral myocarditis is still a challenging disease to diagnose and treat. Nowadays, there are extraordinary diagnostic tests that can help with the diagnostic, including CMR and EMB. Medical management includes the use of ACEi and beta-blockers. Left ventricular assistance devices should be considered in acute severe cases with decompensated heart failure and cardiogenic shock. 
